# Common Arterial Trunk Associated with Functionally Univentricular Heart: Anatomical Study and Review of the Literature

**DOI:** 10.3390/jcdd8120175

**Published:** 2021-12-06

**Authors:** Sami Chatila, Lucile Houyel, Manon Hily, Damien Bonnet

**Affiliations:** 1 Congenital and Pediatric Cardiology Unit, M3C-Necker, Hôpital Necker-Enfants Malades, AP-HP, 75015 Paris, France; chatilasami@gmail.com (S.C.); manon.hily@aphp.fr (M.H.); damien.bonnet1@gmail.com (D.B.); 2 Université de Paris, 75006 Paris, France

**Keywords:** common arterial trunk, univentricular heart, tricuspid atresia, mitral atresia, double inlet left ventricle, atrioventricular septal defect

## Abstract

Common arterial trunk (CAT) is a rare congenital heart disease that is commonly included into the spectrum of conotruncal heart defects. CAT is rarely associated with functionally univentricular hearts, and only few cases have been described so far. Here, we describe the anatomical characteristics of CAT associated with a univentricular heart diagnosed in children and fetuses referred to our institution, and we completed the anatomical description of this rare condition through an extensive review of the literature. The complete cohort ultimately gathered 32 cases described in the literature completed by seven cases from our unit (seven fetuses and one child). Four types of univentricular hearts associated with CAT were observed: tricuspid atresia or hypoplastic right ventricle in 16 cases, mitral atresia or hypoplastic left ventricle in 12 cases, double-inlet left ventricle in 2 cases, and unbalanced atrioventricular septal defect in 9 cases. Our study questions the diagnosis of CAT as the exclusive consequence of an anomaly of the wedging process, following the convergence between the embryonic atrioventricular canal and the common outflow tract. We confirm that some forms of CAT can be considered to be due to an arrest of cardiac development at the stages preceding the convergence.

## 1. Introduction

Common arterial trunk (CAT) is a rare birth defect with an estimated incidence of one birth in 10,000 [1]. This congenital disorder is part of the so-called “conotruncal” heart diseases spectrum, also called cardiac neural crest and second heart field defects, characterized by anomalies in the development and position of the outflow tract, and by the presence of a ventricular septal defect (VSD) related to malalignment or the absence of the development of the outlet septum (outlet VSD) [2]. Associations between CAT and defects of the inlet or apical segments of the heart have been described in the literature, including malformations of the atrioventricular junction and various degrees of ventricular hypoplasia. These anomalies often correspond to a hypoplasia of an atrioventricular valve or to the absence of an atrioventricular connection (mitral or tricuspid atresia). Hitherto, however, these cases have only been described through case reports or short series. The purpose of this study was (1) to describe a series of one patient, five fetal heart specimens, and one fetus with these rare associations of CAT and functionally or anatomically univentricular heart, and (2) to carry out an extensive review of the literature.

## 2. Anatomical Definitions

### 2.1. Common Arterial Trunk

Common arterial trunk is defined as “a congenital cardiovascular malformation in which a single arterial trunk arises from the heart, giving origin sequentially to the coronary arteries, one or more pulmonary arteries, and the systemic arterial circulation” [3]. There is therefore a common ventriculo–arterial junction, with a common arterial valve, above a large outlet juxta–arterial VSD. The different types of CAT are classified according to the dominance of either the systemic of pulmonary components of the common trunk [2,3,4,5].

- CAT with aortic dominance and confluent pulmonary arteries (Type 1–2 of the modified Van Praagh classification).
- CAT with aortic dominance and discontinuous pulmonary arteries (Type 3 of the modified Van Praagh classification). One pulmonary artery originates from the CAT, the other is supplied by the arterial duct or collateral arteries.
- CAT with pulmonary dominance and interruption of the aortic arch (IAA, usually type B of Celoria and Patton [6], between the left carotid artery and the left subclavian artery), or coarctation of the aorta (Type 4 of the modified Van Praagh classification).

### 2.2. Functionally Univentricular Heart

According to the International Society of Nomenclature for Pediatric and Congenital Heart Disease (ICD-11 IPCCC), the term functionally univentricular heart describes a spectrum of congenital cardiac malformations in which the ventricular mass may not readily lend itself to partitioning that commits one ventricular pump to the systemic circulation, and another to the pulmonary circulation [3]. In addition, “a heart may be functionally univentricular because of its anatomy or because of the lack of feasibility or lack of advisability of surgically partitioning the ventricular mass”.

Common lesions in this category typically include double-inlet ventricles, tricuspid atresia, mitral atresia, and hypoplastic left heart syndrome. Unbalanced atrioventricular septal defects (AVSD) with hypoplasia of one ventricle may also be considered functionally univentricular hearts.

## 3. Methods

### 3.1. Data Collection

The data presented here are the result of a systematic review of the available literature, to which we added the analysis of five fetal heart specimens from the M3C-Necker anatomical collection, one fetal echocardiography, and the follow-up of a patient hospitalized in the pediatric cardiology department of Necker–Enfants malades University Hospital. Data for the living patient were obtained from the Necker–Enfants malades and Imagine Institute congenital heart disease database using Dr Warehouse^®^, a full-text clinical data warehouse (CDW) for cohort identification and data extraction [7].

### 3.2. Literature Review

Articles of interest were selected online using the PubMed database. All articles were gathered by a keyword search and by cross referencing previously obtained articles. Articles of interest were found using the following keyword associations: “Univentricular heart”, “Single ventricle”, “Hypoplastic ventricle”, “Ventricular hypoplasia”, “Double inlet ventricle”, “Fontan”, “Tricuspid atresia”, “Mitral atresia”, “Complete atrioventricular canal”, “Unbalanced atrioventricular septal defect”, “Common arterial trunk”, “Arterial trunk “, and “Persistent arterial trunk”. No limitation was included regarding publication date, studied population, or language. SC and LH reviewed the search results independently to select the reports based on the inclusion and exclusion criteria. Chest X-ray, echocardiography, angiography, and CT images were interpreted when available, compared to the authors’ interpretation, and exploited when textual data were unavailable. The literature review was conducted in accordance with the Preferred Reporting Items for Systematic Reviews and Meta-Analyses (PRISMA) guidelines [8]. 

**Figure 1:**
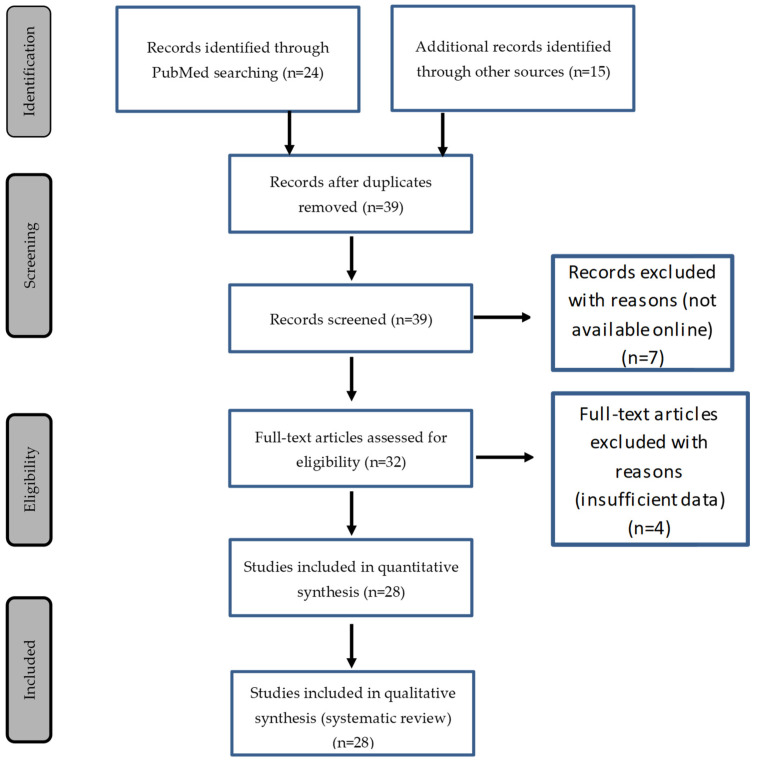
PRISMA (Preferred Reporting Items for Systematic Reviews and Meta-Analyses) flow diagram of the study selection.

Thirty-nine articles whose title and abstract matched the association between a functionally or anatomically univentricular heart and a CAT were collected. Eleven articles were excluded from this list: seven of them were not available online, and four of them presented insufficient data for proper interpretation (Figure 1). Among the selected articles [9,10,11,12,13,14,15,16,17,18,19,20,21,22,23,24,25,26,27,28,29,30,31,32,33,34,35,36], only one included a series of five cases [32], the others were case reports of one or two patients (Table 1). Among the functionally univentricular hearts, we decided to include only hearts with severe right or left ventricular hypoplasia, including unbalanced AVSDs. Three cases of complete AVSD with balanced ventricles who were not eligible for a biventricular repair due to abnormal valve attachments in the LV-to-aorta pathway were excluded from the cohort [32]. 

### 3.3. Heart Specimens

Five fetal heart specimens (Cases 33 to 37, Table 2) were extracted from the M3C-Necker anatomic collection of fetal hearts by MH, and were analyzed by LH, using the segmental analysis method as described by Van Praagh [37].

### 3.4. Fetal Echocardiography

A diagnosis of CAT type 1 associated with severe tricuspid and RV hypoplasia was made at 25 weeks of gestation (WG) on a fetal echocardiography (Case 38, Table 2). Six weeks later, the pregnancy was ongoing, without any pejorative event. 

### 3.5. Case Report

A prenatal diagnosis of tricuspid atresia associated with a CAT type 1 was made at 20 WG (Case 39, Table 2). Pregnancy was obtained after in vitro fertilization and intracytoplasmic sperm injection. No chromosome 22q11 microdeletion was identified by FISH and the CGH array on the amniotic fluid was normal.

**Figure 2:**
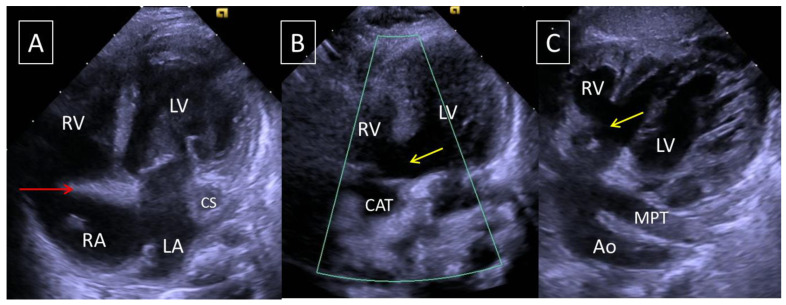
Case 39 (Table 2) with tricuspid atresia and common arterial trunk type 1: echocardiographic views. (**A**): Four-chamber apical view. Red arrow: absence of right atrioventricular connection (tricuspid atresia). CS, dilated coronary sinus; LA, left atrium; LV, left ventricle; RA, right atrium; RV, right ventricle. (**B**): Five-chamber apical view. The common arterial trunk overrides the ventricular septum, above a large outlet ventricular septal defect (yellow arrow). CAT, common arterial trunk; LV, left ventricle; RV, right ventricle. (**C**): Subcostal view. Common arterial trunk type 1. Yellow arrow, outlet VSD; Large outlet VSD. Ao: Aorta; MPT: Main pulmonary trunk.

A 3010 g male child was delivered at term. He adapted well to extrauterine life. Initial echocardiography found atrial situs solitus, levocardia, D-loop ventricles, normal pulmonary venous returns, a left superior caval vein draining in the coronary sinus, a large ostium secundum type atrial septal defect with a right to left shunt, and a hypoplastic right ventricle (RV) with absent right atrioventricular connection. The left ventricle (LV) was fully developed with a normal size mitral valve with mild regurgitation. A large CAT overrode the ventricular septum above a large outlet juxta-arterial VSD (Figure 2). From the CAT originated successively the coronary arteries, a short pulmonary arterial trunk with two normal size pulmonary arteries, and a left aortic arch. There was no arterial duct. The patient was discharged after leaving the maternity ward. Furosemide therapy was initiated at 2 weeks of age because of clinical signs of excessive pulmonary blood flow.

At the age of 1 month, the patient was admitted to our department for surgical management. The first intervention consisted of a disconnection of the pulmonary arteries and the creation of a 4 mm Gore-Tex systemic-to-pulmonary shunt (modified Blalock-Taussig-Thomas (BTT) anastomosis). The child was extubated, weaned from inotropic support on the fourth postoperative day, and discharged from the hospital on the ninth postoperative day.

At 3 years of age, catheterization showed mean pulmonary artery pressure of 14 mmHg with a good function of the left ventricle (end-diastolic left ventricular pressure 10 mmHg). The child underwent a non-fenestrated total cavopulmonary connection with extracardiac conduit and pulmonary bifurcation repair with a Gore-Tex patch. He was quickly extubated and weaned from inotropic support. The postoperative course was complicated by a second chylothorax, which regressed after a 6 week fat free diet. He was discharged home at day 5, under diuretic treatment and Aspirin.

At the age of 5 months, heart catheterization was performed. The mean right and left mean pulmonary pressures were 10 mmHg. The BTT shunt was patent, and angiographies showed normal size left and right pulmonary arteries. Bilateral partial cavopulmonary connection was performed, with a section of azygos and hemi-azygos veins. The aortic oxygen saturation at the end of the procedure was 90%. The child was quickly extubated and the inotropes were weaned. The postoperative course was complicated by a chylothorax treated with a fat-free diet for 6 weeks.

At the last follow-up visit at the age of 3.5 years, the patient was still in good condition. Oxygen saturation was normal. He was treated with aspirin and was schooled normally.

### 3.6. Statistical Analysis

Statistical analysis was performed with Statview 5.0. The statistics were descriptive and expressed as percentages. Fisher’s exact test for was used for comparison between the two major groups of hypoplastic RV and hypoplastic LV. A value of p < 0.05 was considered statistically significant.

## 4. Results

The anatomical description of each case has been summarized in Table 1 (review of the literature, cases 1 to 32) and Table 2 (cases 33 to 39). The distribution of the anatomical characteristics of the cohort is summarized in Table 3.

Among these 39 cases, four types of functionally or anatomically univentricular hearts were found in association with CAT: 

### 4.1. Tricuspid Atresia and Hypoplastic Right Ventricle

**Figure 3:**
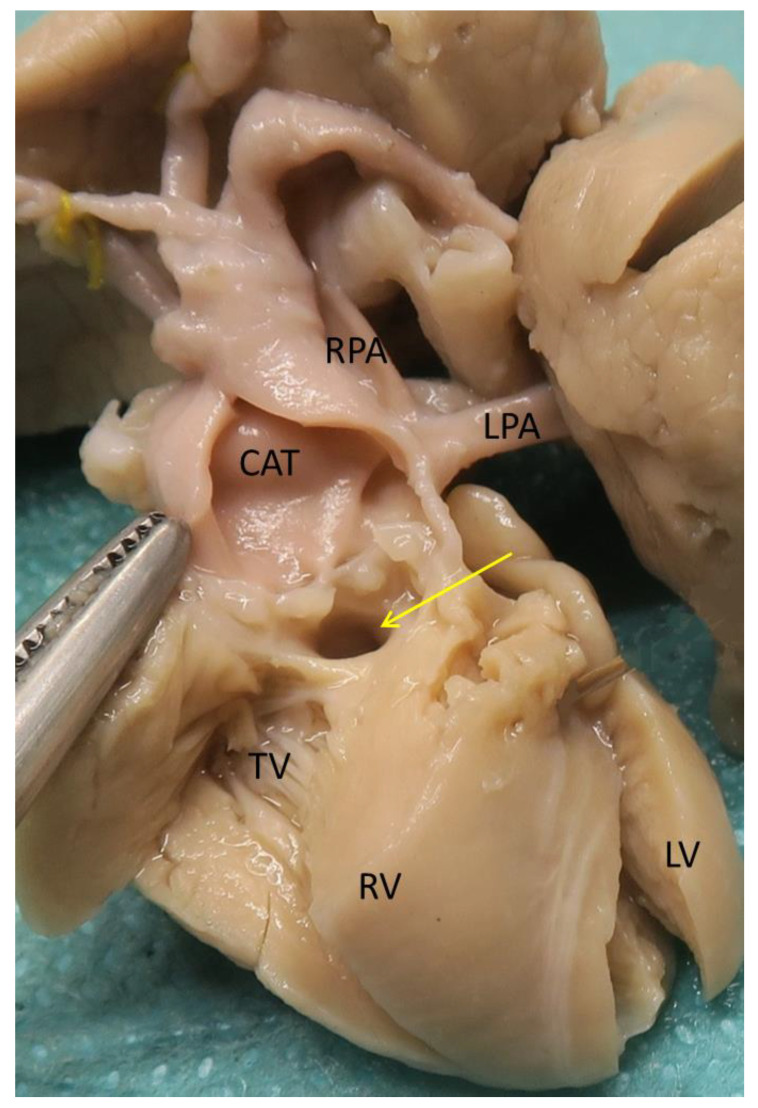
Heart specimen with common arterial trunk type 1 and hypoplastic right ventricle (Table 3, Case 33). The truncal valve is quadricuspid. Yellow arrow, outlet ventricular septal defect; CAT, common arterial trunk; LPA, left pulmonary artery; LV, left ventricle; RPA, right pulmonary artery; RV, right ventricle; TV, tricuspid valve.

Tricuspid atresia and hypoplastic RV were found in 16/39 cases (41%). Tricuspid atresia was observed in 11 cases and RV hypoplasia in 5. One of the cases was associated with Di George syndrome. There was no situs anomaly or heterotaxy syndrome. Systemic venous returns were always normal, except for one case of persistent left superior caval vein in the coronary sinus (Case 39). One case was associated with a partial anomalous pulmonary venous return (APVR) of the left upper pulmonary vein in the innominate vein (Case 3). The VSD was always of the outlet type (Figure 3), adjacent to the truncal valve (Table 3), except in one patient without VSD (Case 2). CAT was most often type 1 (12 cases, Figure 3), type 2 in two cases, type 3 in one case, and type 4 in one case with IAA type B. A right aortic arch was described in three cases, including one with associated Di George syndrome (Cases 9 to 11). In two cases there was a coronary anomaly: atresia of the right coronary ostium with RV perfusion by myocardial sinusoids in Case 2, and left coronary orifice within the right sinus of Valsalva in Case 1. One case with tricuspid atresia had a left juxtaposition of the atrial appendages (Case 12). 

### 4.2. Mitral Atresia and Left Ventricle Hypoplasia

**Figure 4:**
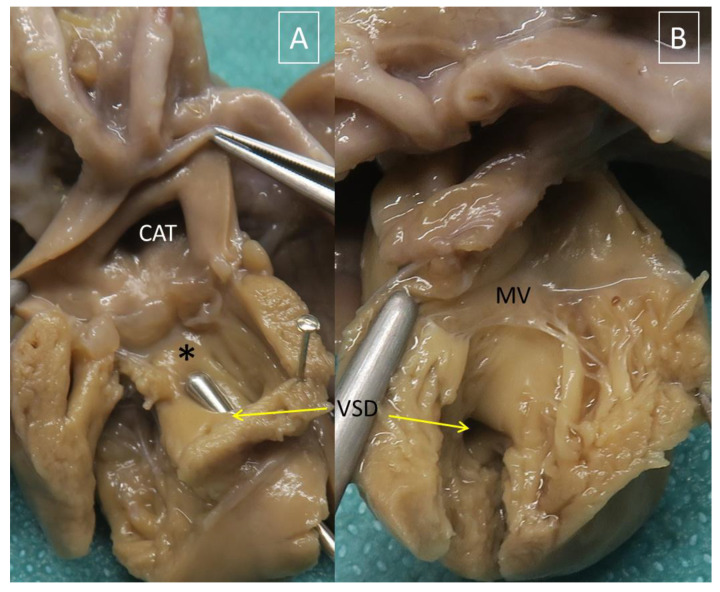
Heart specimen with common arterial trunk type 4, aortic coarctation and hypoplastic left ventricle (Table 3, Case 35). (**A**): view from the right ventricle. The common arterial trunk (CAT) is entirely above the right ventricle and there is a subarterial conus (asterisk). (**B**): view from the left ventricle. There is a large mid-muscular ventricular septal defect (yellow arrow).

**Figure 5:**
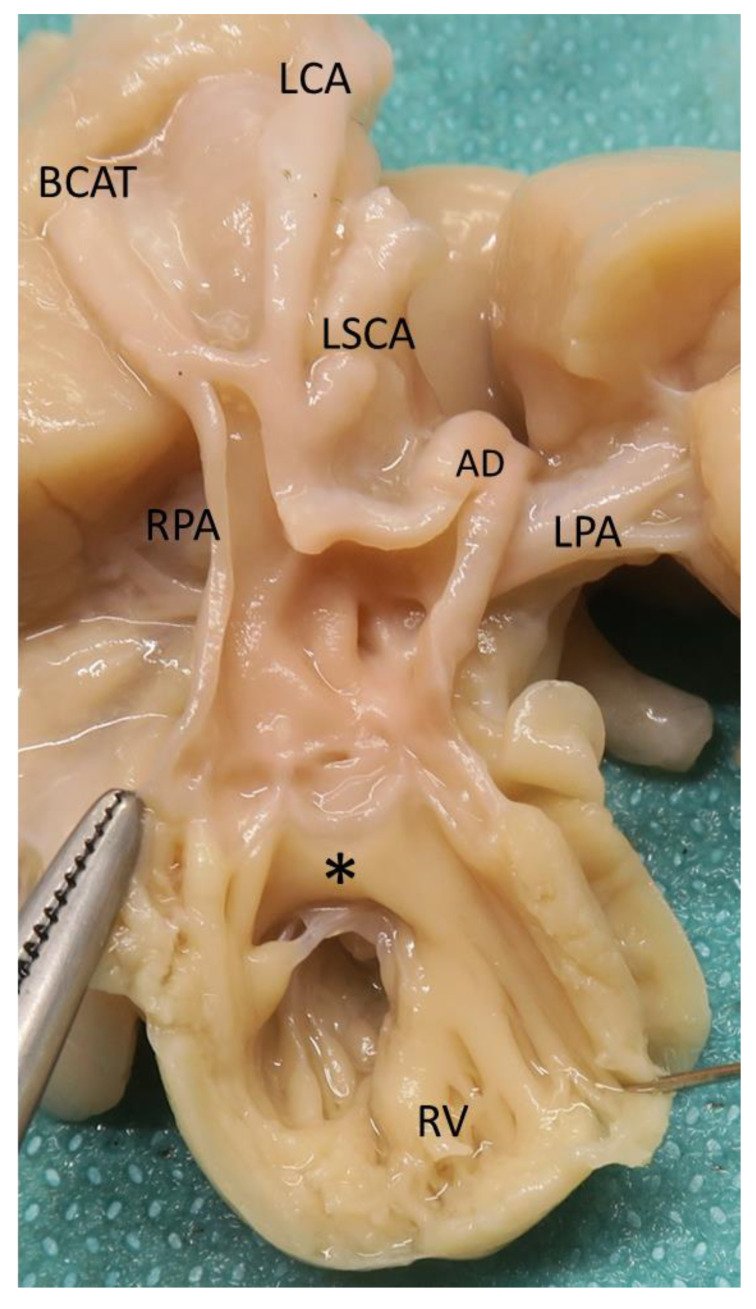
Heart specimen with common arterial trunk type 4, interruption of the aortic arch type A, left aortic arch, and hypoplastic left ventricle (Table 3, Case 36). View from the right ventricle. The common arterial trunk (CAT) is entirely above the right ventricle and there is a subarterial conus (asterisk). AD, arterial duct; BCAT, brachiocephalic arterial trunk; LCA: Left carotid artery; LPA, left pulmonary artery; LSCA, left subclavian artery; RPA, right pulmonary artery; RV, right ventricle.

Mitral atresia or LV hypoplasia was found in 12/39 cases (31%). Mitral atresia was observed in six cases and LV hypoplasia in six. One case was associated with Di George syndrome (Case 17). None of the cases had situs or looping anomalies. Systemic venous returns were normal except for four cases of persistent left superior caval vein, draining in the coronary sinus in three (Cases 18,36,37), and the site of drainage being not specified in one (Case 16). Two cases of AVPR were found: one had total APVR to the coronary sinus (Case 37) and the other was partial (right upper pulmonary vein in the right superior caval vein, Case 13). An intact ventricular septum was found in seven cases, and a subarterial conus was described in eight cases (Figure 4). There was an outlet VSD in three cases, a mid-muscular VSD in one case, and the VSD type was not described in one case. The truncal valve was described as originating from the RV in nine cases and overriding the ventricular septum in three cases. The CAT was type 1 or 2 in the majority of cases (8/12, 66%) and type 4 in four cases. In those four last cases, one had hypoplasia of the transverse aorta, two had IAA type A (Figure 5), and one IAA type B. One of these 12 cases had a double aortic arch, and one a right aortic arch, both unrelated to Di George syndrome. An abnormal coronary origin was present in five cases (two cases with hypoplasia of the left coronary orifice, one case with high take-off of the left coronary artery, just above the commissure between the “right and left coronary cusps”, one case with right coronary orifice within the posterior sinus, and one case with a single coronary artery originating from the innominate artery).

### 4.3. Double Inlet Left Ventricle:

This is an extremely rare association as only two cases were described in the literature (Cases 22 and 23). Both cases were in situs solitus with D-loop ventricles. Situs of other organs was not described. Left juxtaposition of the atrial appendages was present in both cases and dextrocardia in 1. A superior left caval vein drained in the left atrium in Case 22. The CAT was type 1 and 2, and emerged from the left ventricle in both. There was a muscular VSD in Case 22, and a large outlet VSD in Case 23.

### 4.4. Unbalanced Complete AVSD

We found nine cases of unbalanced complete AVSD associated with CAT reported in the literature (23% of all cases). Of these, seven had a hypoplastic left ventricle, while the other two had right ventricular hypoplasia, both of them having Di George syndrome (Cases 24 and 25). Case 30 was associated with atrial situs inversus, dextrocardia, and common atrium, without heterotaxy. Two of the cases were associated with a persistent superior left caval vein, without a description of its connection. The VSD was of the inlet type in seven, restrictive in four of them, and muscular (bulbo-ventricular foramen) in two. The CAT was type 1 in six cases and type 2 in three cases. When the right ventricle was dominant, CAT originated exclusively from the RV in 5/7 cases. A patient with type 2 CAT also had left pulmonary artery hypoplasia. The aortic arch was right-sided in five cases, including both cases associated with Di George’s syndrome.

### 4.5. Global RV and LV Hypoplasia

We decided to group together those cases of the four groups above with RV hypoplasia (*n* = 20) and those with LV hypoplasia (*n* = 19) (Table 3). The ventricular septum was intact in only one case of Group 1 (5%) vs. seven in Group 2 (37%, *p* < 0.02). When present and when its type was specified, the VSD was of the outlet type in 16/18 cases of Group 1 (89%) vs. 3/11 patients of Group 2 (27%, *p* = 0.002). The common arterial trunk originated exclusively from the RV in two cases of Group 1 (10%) vs. 14 of Group 2 (74%, *p* = 0.0001). The last significant difference between the two groups was that a subarterial conus was found almost exclusively in Group 2 (5% vs. 42%, *p* < 0.01).

## 5. Discussion

So-called “conotruncal” defects consist of a spectrum of outflow tract anomalies due to an abnormal rotation and/or septation of the outflow tract. The differences in “conotruncal” heart defect phenotypes can be explained by the degree of rotation of the aortic valve and the position of the outlet septum relative to the outflow tract of the two ventricles [38,39,40]. The persistence of the common arterial trunk corresponds to a very early interruption of the mechanism of rotation and the septation of the outflow tract [41]. The absence of colonization of the outflow endocardial cushions by neural crest cells leads to a lack of fusion of the outflow tract cushions, explaining the non-separation of the two great vessels and the presence of a common truncal valve. The lack of addition of myocardial cells from the anterior second heart field to the developing outflow tract results in an absence of development of the outlet septum, leading to a large outlet juxta-arterial VSD, and in the lack of elongation of the outflow tract, preventing normal wedging of the aortic valve between mitral and tricuspid valve, with an outflow tract that is shorter than normal [42,43,44]. Comparatively, a lack of rotation of the developing outflow tract with normal arterial septation leads to the various malalignment defects (double outlet right ventricle (DORV) with subaortic, doubly committed or subpulmonary VSD, tetralogy of Fallot and variants, overriding aorta, IAA type B). All these defects include an outlet VSD due to the malalignment and absence of fusion of the outlet septum with the rest of the ventricular septum [40]. 

Common arterial trunk is most often an anomaly of the outflow tract only, the most common association being aortic arch anomalies and 22q11.2 deletion syndrome. The incidence of 22q11.2 deletion is estimated to be 32%, and is higher in types 3 and 4 and when there are associated aortic arch anomalies such as a right aortic arch or aberrant subclavian artery [45]. Association to anomalies of other segments of the heart, such as atrioventricular septal defect, or ventricular hypoplasia, is exceptional [46,47]. Rare associations with anomalous pulmonary venous return have also been reported [48]. This series confirms the rarity of anatomically or functionally univentricular hearts in association with CAT. 

### 5.1. Common Arterial Trunk Associated with Functionally Univentricular Heart has Specific Anatomic Characteristics Compared to Isolated CAT

Contrarily to the usual CAT where an outlet juxta–arterial VSD is an integral part of the phenotype except in very rare cases [49], an intact ventricular septum was found in 8/39 cases (20.5%) in our series. The absence of VSD was almost unique to left ventricular hypoplasia, except for one case with hypoplasia of RV and the tricuspid valve (Table 3). In all other cases with a hypoplastic RV, the VSD was of the usual type, outlet juxta–arterial, located between the two limbs of the septal band and adjacent to the truncal valve, except in the two cases with unbalanced AVSD and hypoplastic RV in whom the VSD was muscular [32]. Conversely, only three cases with a hypoplastic LV had an outlet VSD, described as tiny in one. In addition, a subarterial conus—exceptional in classical CAT—was found in 8/19 cases (42%) with a hypoplastic LV (6 of them had no VSD), while a small subarterial conus was found in only one case with hypoplastic RV and tricuspid hypoplasia (Table 3). Interestingly, in all those nine cases with a subarterial conus, the CAT arose exclusively above the RV (Table 1 and Table 2).

The presence in these nine cases of a CAT emerging entirely from the RV above a subarterial conus suggests an anatomy similar to the double outlet right ventricle, corresponding to group II of Van Praagh’s classification [50]. This type of double outlet right ventricle is the consequence of an arrest of heart development at the early looping stage, before the convergence of the inlet and outlet segments of the heart, and is associated with a “non-committed” muscular or inlet type of VSD, often with hypoplasia of the left ventricle and hypoplasia or atresia of the mitral valve. In our cohort, this anatomy suggestive of double outlet right ventricle is associated with the common ventriculo–arterial junction due to a failure of aorticopulmonary septation and arterial valve formation, while the outlet septum is well formed and therefore results in the presence of a complete subarterial infundibulum. This association would plead in favor of a different mechanism for CAT morphogenesis from what is observed in the isolated “conotruncal” type of CAT, where there is no subarterial conus and an outlet juxta–arterial VSD.

There were no differences in our cohort regarding the anatomic type of CAT and the number of truncal leaflets compared to usual forms of CAT. The truncal valve was tricuspid in 69% of cases, quadricuspid in 23%, and bicuspid in 8%. Common arterial trunk was type 1 or 2 in 85%, type 3 in only 1 case (2%), and type 4 in 5 (13%). These figures are comparable to those described in other series [49].

Coronary anomalies are frequent in CAT, and are up to 87% in anatomic series [39]. These are mostly anomalies of the position, size, and shape of the coronary orifices, more frequently the left coronary orifice [39]. In our series, the rate of coronary anomalies was much lower (7/39, 18%). All were anomalies of the orifices, except in two cases. The first one, described by Zeevi and al. [10], had a coronary abnormality very similar to those encountered in pulmonary atresia with an intact ventricular septum. It is indeed the only case in this series with a hypoplastic RV and no VSD. The CAT originated exclusively above the left ventricle, the tricuspid valve was hypoplastic but patent, with suprasystemic RV pressure, and the right coronary orifice was atretic with multiple ventriculo–coronary connections. The second one, with mitral atresia, no VSD and the CAT exclusively above the right ventricle, had a single coronary artery originating from the innominate artery [29].

### 5.2. The Phenotype of Functionally Univentricular Hearts Associated with CAT Is Different from Their Classical Forms Associated with Separate Ventriculo–Arterial Junctions 

In the study, we chose to include all types of functionally or anatomically univentricular hearts that were found in association with CAT. In the whole series, there were almost exactly as many cases with hypoplastic RV as those with hypoplastic LV. However, the LV was hypoplastic in the majority of cases with unbalanced AVSD (78%).

Tricuspid atresia associated with common arterial trunk was included in the classification of tricuspid atresia by Tandon and Edwards in 1974, despite the rarity of this association [51]. This classification, modified by Rao in 1980 [52], is based on the type of ventriculo–arterial connection, and on the presence or absence of pulmonary stenosis or atresia. A striking difference between the intracardiac anatomy of our cases with CAT and tricuspid atresia or hypoplasia, and those with classic forms of tricuspid atresia is that the VSD in our cases is not the persisting primary interventricular foramen or bulbo-ventricular foramen with muscular borders, but an outlet juxta–arterial VSD, like in usual “conotruncal” forms of CAT. The fact that the VSD in tricuspid atresia almost always has muscular borders could thus be explained not only by the persistence of the primary interventricular foramen [53], but also by the septation of the outflow tract itself, which involves fusion and muscularization of the outflow tract endocardial cushions to produce the subarterial conus. This does not seem to be the case in mitral hypoplasia or atresia, as the VSD is of the outlet type in only 16.7% of cases. 

It is of note that the association of tricuspid atresia and CAT could be considered as an arrest in heart development at a very early stage. Indeed, the embryonic outflow tract lies entirely above the RV until the convergence between the embryonic atrioventricular canal and the common outflow tract, leading to correct alignment between the atrial and ventricular septal, and the beginning of the rotation of the aortic valve towards the left ventricle [53]. However, in this series, only two cases with hypoplastic RV had a CAT located entirely above the hypoplastic RV (Cases 3 and 33). Therefore, there might be a variation in the degree of convergence, which influences the final position of the CAT relative to the ventricular septum.

Surprisingly, the association between the double inlet left ventricle and CAT is very rare, with only two cases described in the literature. The double inlet left ventricle corresponds to a very early interruption in heart morphogenesis, before the establishment of the right atrioventricular junction and the convergence stage. Indeed, the primitive ventricle is the morphologically left ventricle. If cardiac development is interrupted immediately after cardiac looping (early looping stage), there will be a malalignment of the atrial and ventricular septa and the two atrioventricular valves will open in the morphologically left ventricle. The segmental analysis is most often {S,L,L}, but the two cases described in our cohort were {S,D,D}. The VSD in double-inlet left ventricle is muscular, tends to be restrictive, and corresponds to the primitive interventricular communication or bulbo–ventricular foramen [53]. However, in one of the two cases of our cohort the VSD was of the outlet type.

The morphogenesis of double inlet left ventricle with CAT could thus correspond to a global arrest of cardiac development at a very early stage. This could be supported in our two cases by the presence of the left juxtaposition of the atrial appendages, with dextrocardia in one of them. However, while an arrest of development of the ventricles at this stage might explain the double inlet left ventricle and also tricuspid atresia, the fact that there are almost always two distinct great arteries in these congenital cardiac defects suggests that the outflow tract develops independently from the ventricular segment in the majority of cases. Total arrest of cardiac development at the early looping stage, including aortopulmonary septation, appears to be extremely rare, for a still unknown reason.

Complete unbalanced ASVD associated with CAT is a complex and rare heart disease related to both an outflow tract septation defect (related to cardiac neural crest and anterior second heart field) and an atrioventricular septation defect (related to posterior second heart field). This dual origin has also been evoked in the rare association of CAT and anomalous pulmonary venous return [48].

Embryologically, it is possible to distinguish two types of AVSD: “early” AVSD associated with heterotaxy syndrome, resulting from persistence of the embryonic atrioventricular canal, and the later, isolated AVSD, which results from a defective atrioventricular septation related to a lack of growth of the vestibular spine, derived from the posterior heart field [54,55,56]. It must be underlined that none of our cases displayed heterotaxy features, and only one case with unbalanced AVSD had atrial situs inversus, ruling out disturbed laterality as a major determinant of the association between CAT and a functionally univentricular heart. This is in accordance with the very low rate of common arterial trunk associated with laterality defects in the National Birth Defects Prevention Study: 0.8% in overall laterality defects and 1.1% in heterotaxy [57]. A few cases of CAT in the setting of heterotaxy with right isomerism have been reported, with or without ventricular hypoplasia [47,58,59]. Interestingly, Pitx2abc null mice mutants display right atrial isomerism associated with a common arterial trunk, indicating that altered left-right signaling at the venous pole can be associated with an abnormal signaling in the cardiac neural crest, leading to CAT [60].

## 6. Conclusions

Our study questions the phenotype “CAT” as a defect affecting exclusively the development and septation of the outflow tract. We can hypothesize that in the rare occurrence of association with abnormalities in other cardiac segments, the visible phenotype of CAT is not exclusively the consequence of an anomaly of wedging (“late” CAT), but could also be due to a disturbance or interruption of heart development at earlier stages, prior to convergence between the embryonic atrioventricular canal and the common outflow tract (“early” CAT). In this case, the “early” CAT would be homoplastic to the “late” CAT, in the sense that they share the same phenotype regarding the anatomy of the unique vessel arising from the heart, although the developmental defect that underlies this phenotype is different. In other words, CAT can be considered not only as a specific congenital heart defect, but as one of the abnormal phenotypes of the outflow tract of the heart with a largely predominant association with outlet VSD, normal size left and right ventricles and normal convergence (usual “late” forms of CAT), and at a much lower frequency with earlier and more complex intracardiac anatomies (“early” forms of CAT). Finally, we found no heterotaxy in this series, confirming that the prevalence of the phenotype “CAT” in heterotaxy is indeed very low [56]. 

The rare associations of CAT with a variety of underlying early anomalies of cardiac development leading to functionally univentricular heart illustrate the fact that some cardiac defects involving a segment of the heart (segmental phenotypes) can be observed in association with different anomalies of the other segments. Identifying CAT as a congenital heart defect belonging to the group of outflow tract malformations proceeds of the idea of developmental or phylogenetic relationships between these defects—they belong to the same path of abnormal development, the same clade. This is the usual cladistic approach. Identifying CAT as a physical attribute in the setting of a non-limited spectrum of cardiac defects, that is considering it as one type of ventriculo–arterial connection among others and not only as a congenital heart disease by itself, and naming it CAT based on phenotypic similarities, is a phenetic approach. This approach does not necessarily reflect genetic similarity or evolutionary relatedness, and is based only on observable characteristics, here the total absence of septation of the outflow tract (CAT), which can occur with all types of atrioventricular connections and all degrees of development of the ventricles. This new approach can be applied to all varieties of segmental phenotypes in congenital heart diseases, opening new perspectives in the comprehension and analysis of these congenital anomalies.

## Figures and Tables

**Table 1:** Anatomical characteristics of cases 1 to 32.

Authors Case Number	Chromosomal Abnormality	FUVH Type	VSD	CAT Type/ Origin	Truncal Valve Nb Leaflets	Aortic Arch	Anomalies
Fujimoto et al. [9] Case 1		Hypoplastic RV	Large outlet	4	4	Left	IAA type B, left coronary ostium from right sinus
Zeevi et al. [10] Case 2		Hypoplastic RV	No VSD	1 from LV	2	Left	No ASD, mitral stenosis, atretic right coronary ostium with retrograde sinusoid filling
Gonzalez-Lopez et al. [11] Case 3		Tricuspid atresia	Outlet	1 from RV	3	Left	LUPV in retro aortic innominate vein
Rao et al. [12] Case 4		Tricuspid atresia	Outlet	1	3	Left	Hypoplastic thymus, right thumb and hemivertebrae, narrow PA branches
Roldan et al. [13] Case 5		Tricuspid atresia	Large outlet	2	US	US	
Malec et al. [14] Case 6		Tricuspid atresia	Outlet	1	US	Left	Restrictive ASD
Numata et al. [15] Case 7		Tricuspid atresia	Outlet	1	US	US	
Sreeram et al. [16] Case 8		Tricuspid atresia	Outlet	1	US	Left	
Alva et al. [17] Case 9	Di George	Tricuspid atresia	Outlet	1 from LV	US	Right	
Areias et al. [18] Case 10		Tricuspid atresia	Outlet	3	3	Right	LPA from left-sided duct
Sharma et al. [19] Case 11		Tricuspid atresia	Large outlet	1 from LV	3	Right	Two OS ASD
Diogenes et al. [20] Case 12		Tricuspid atresia	Large outlet	2	2	Left	Large ASD, L-JAA
Hoashi et al. [21] Case 13		Hypoplastic LV	Large outlet	2	4	Right	RUPLV in right SCV
Marathe et al. [22] Case 14		Hypoplastic LV	No VSD	4 from RV	3	Left	IAA type B
Murdison et al. [23] Case 15		Hypoplastic LV	No VSD	1 From RV	3	Left	Subarterial conus, only 2 brachiocephalic arteries, RCA origin from posterior cusp
Imai et al. [24] Case 16		Hypoplastic LV	US	2	US	Double	LSVC
Michelfelder et al. [25] Case 17	Di George	Mitral atresia	No VSD	1 From RV	3	Left	Restrictive ASD, Subarterial conus
Rice et al. [26] Case 18		Mitral atresia	Outlet	1 From RV	4	Left	LSCV to CS, Subarterial conus
Jacobs et al. [27] Case 19		Mitral atresia	Tiny outlet	1	US	US	
Alves et al. [28] Case 20		Mitral atresia	No VSD	4 from RV	3	Left	Subarterial conus, IAA Type A, LCA ostium supracommissural
Cree et al. [29] Case 21		Mitral atresia	No VSD	2 from RV	3		Subarterial conus, single coronary artery from inominate artery
Shaddy et al. [30] Case 22		Double inlet LV	US	1	US	Left	Situs Solitus {SDD}, LSCV to LA, L-JAA
Paris et al. [31] Case 23		Double inlet LV	Large outlet	2	3	Left	Situs Solitus {SDD}, dextrocardia, L-JAA
He et al. [32] Cases 24 and 25	Di George	uAVSD (hypo LV)	Large inlet extending to outlet	2	3	Right	LSCV
	Di George	uAVSD (hypo LV)	Large inlet extending to muscular septum	2	4	Right	
Panwar et al. [33] Cases 26 and 27		uAVSD (hypoLV)	Restrictive inlet	1 from RV	US	US	
		uAVSD (hypoLV)	Restrictive inlet	1 from RV	US	Right	
Tripathi et al. [34] Cases 28 and 29		uAVSD (hypoLV)	Restrictive inlet	1 from RV	3	Right	
		uAVSD (hypoLV)	Restrictive inlet with muscular extension	1 from RV	US	Left	LSCV
Kumar et al. [35] Case 30		uAVSD (hypoLV)	Inlet	2 from RV	3	Right	Situs inversus, dextrocardia, common atrium
Shapiro et al. [36] Cases 31 and 32		uAVSD (hypoRV)	Muscular (BVF)	1	US	Left	PA trunk stenosis
		uAVSD (hypoRV)	Muscular (BVF)	1 from LV	4	Left	

AoA, aortic arch; ASD, atrial septal defect; BVF, bulboventricular foramen; CAT, common arterial trunk; CS, coronary sinus; FUVH, functionally univentricular heart; IAA, interrupted aortic arch; LCA, left coronary artery; LSCV, left superior caval vein; L-JAA, left juxtaposition of atrial appendages; LUPV, left upper pulmonary vein; LV, left ventricle; OS, Ostium secundum; RCA, right coronary artery; RV, right ventricle; SV, single ventricle; uAVSD, unbalanced atrioventricular septal defect; US, unspecified; VSD, ventricular septal defect.

**Table 2:** Anatomic characteristics of cases 33 to 39.

Case Number	FUVH Type	VSD	CAT Type/ Origin	Truncal Valve Nb Leaflets	Aortic Arch	Anomalies
33 (HS)	Hypoplastic RV	Outlet VSD	1 from RV	4	Left	Subarterial conus
34 (HS)	Hypoplastic RV	Outlet VSD	1	3	Left	Very large ASD
35 (HS)	Mitral atresia	No VSD	2 from RV	3	Left	Subarterial conus, supra commissural RCA ostium, narrow LCA ostium
36 (HS)	Mitral hypoplasia	Large muscular VSD	4 from RV	3	Left	Subarterial conus, narrow LCA ostium, LCSV to CS, Hypoplastic horizontal Ao
37 (HS)	Hypoplastic LV	No VSD	4 from RV	3	Left	Subarterial conus, Type A IAA, LSCV to CS, TAPVR in CS
38 (fetus)	Hypoplastic RV	Outlet	1 from LV	US	US	
39 (patient)	Tricuspid atresia	Large outlet	1	3	Left	LSCV to CS

Abbreviations: Ao, Aorta; ASD, atrial septal defect; CAT, common arterial trunk; CS, coronary sinus; HS, heart specimen; IAA, interrupted aortic arch; LCA, left coronary artery; LSCV, left superior caval vein; LV, left ventricle; RCA, right coronary artery; RV, right ventricle; TAPVR, total anomalous pulmonary venous return; FUVH, univentricular heart; VSD, ventricular septal defect.

**Table 3:** Distribution of the anatomical characteristics of the cohort.

	Tricuspid Atresia and HypoRV N = 16	Mitral Atresia and HypoLV N = 12	DILV N = 2	Unbalanced AVSD N = 9	Hypoplastic RV, Global N = 20	Hypoplastic LV, Global N = 19	p
Systemic venous return							
LSCV to CS	1	3	0	0	1	3	
LSCV to LA	0	0	1	0	1	0	
LSCV (unspecified)	0	1	0	2	0	3	
Pulmonary venous return							
PAPVR	1	1	0	0	1	1	
TAPVR	0	1	0	0	0	1	
Ventricular septal defect							
No VSD	1	7	0	0	1 (5%)	7 (37%)	<0.02
Outlet	15	3	1	0	16 (89%)	3 (27%)	<0.002
Inlet	0	0	0	7	0	7	
Muscular	0	1	0	2	2	1	
Unspecified	0	1	1	0	1	1	
Type CAT							
1	12	4	1	6	15	8	
2	2	4	1	3	3	7	
3	1	0	0	0	1	0	
4	1	4	0	0	1	4	
From RV	2	9	0	5	2 (10%)	14 (74%)	0.0001
From LV	3	0	0	1	3	1	
Truncal valve							
Bicuspid	2	0	0	0	2	0	
Tricuspid	6	8	1	3	7	11	
Quadricuspid	2	2	0	2	3	3	
Unspecified	6	2	1	4	7	6	
Subarterial conus	1 (tiny)	8	0	0	1 (5%)	8 (42%)	<0.01
Coronary artery anomalies	2	5	0	0	2	5	
Aortic arch							
Left	10	9	2	3	14	12	
Right	3	1	0	5	3	6	
IAA type A	0	2	0	0	0	2	
IAA type B	1	1	0	0	1	1	
AoA hypoplasia	0	1	0	0	0	1	
Double AoA	0	1	0	0	0	1	
Unspecified	3	1	0	1	2	2	
L-JAA	1	0	2	0	3	0	

AoA, aortic arch; CAT, common arterial trunk; CS, coronary sinus; IAA, interrupted aortic arch; LA, left atrium; L-JAA, left juxtaposition of the atrial appendages; LSCV, left superior caval vein; PAPVR, partial anomalous pulmonary venous return; RV, right ventricle; TAPVR, total anomalous pulmonary venous return; VSD, ventricular septal defect.

## Data Availability

The data presented in this study are available on request from the corresponding author.
